# Healing Effects of Music, Healing Frequencies, and Binaural Beats for Traumatic Brain Injury and Stroke: A Review

**DOI:** 10.7759/cureus.104261

**Published:** 2026-02-25

**Authors:** Eric Whitney, Jen-Yeu Wang, Jack Haslett, Ajay Ramnot, Javed Siddiqi

**Affiliations:** 1 Neurosurgery, Arrowhead Regional Medical Center, Colton, USA; 2 Neurosurgery, Desert Regional Medical Center, Palm Springs, USA; 3 Neurosurgery, California University of Science and Medicine, Colton, USA

**Keywords:** binaural beats, healing frequencies, music therapy, neuroplasticity, stroke, stroke rehabilitation, tbi, traumatic brain injury

## Abstract

Traumatic brain injury (TBI) and stroke are leading causes of mortality and long-term disability worldwide, necessitating innovative rehabilitation strategies. This review synthesizes current research on the therapeutic effects of music, healing frequencies, and binaural beats in enhancing recovery outcomes for TBI and stroke patients. Music therapy has been shown to improve motor function, cognitive recovery, and emotional well-being through mechanisms such as rhythmic auditory stimulation, neuroplasticity facilitation, and emotional engagement. Healing frequencies, including 432 Hz and 528 Hz, are reported to reduce inflammation, promote cellular repair, and improve neuroplasticity. Binaural beats have demonstrated the potential to modulate brainwave activity, enhance relaxation, and support cognitive and emotional rehabilitation. Physiological mechanisms include the activation of sensorimotor pathways, synchronization of neural networks, and the release of neurotrophic factors. While existing evidence highlights the significant promise of these interventions, challenges such as methodological variability and limited large-scale studies underscore the need for further research. The integration of auditory therapies into multimodal rehabilitation protocols offers a cost-effective and patient-centered approach to improving recovery outcomes and quality of life for individuals affected by TBI and stroke.

## Introduction and background

Traumatic brain injury (TBI) and stroke are among the most significant global health challenges, contributing to high mortality and long-term disability rates. TBI affects approximately 69 million individuals annually, often leaving survivors with severe neurological impairments [1]. Preservation of auditory pathways during early recovery phases is of utmost importance, as patients with disorders of consciousness who retain the ability to follow commands within 28 days after injury demonstrate significantly better functional outcomes within one year [2]. Similarly, stroke causes an estimated 6.6 million deaths annually and affects 101.5 million people worldwide, underscoring the urgent need for effective rehabilitation strategies [3].

Historically, music has been recognized for its healing properties. Ancient Greek and Egyptian physicians used flutes and lyres to promote digestion, alleviate mental disturbances, and induce sleep [4]. In the modern era, Diogel's pioneering 1880 study at Salpêtrière Hospital in Paris provided empirical evidence of music's therapeutic effects, demonstrating reductions in blood pressure and heart rate while promoting parasympathetic nervous system activity [4]. Contemporary research has further substantiated music therapy's benefits for stroke and TBI patients. A meta-analysis concluded that music therapy significantly enhances motor recovery in post-stroke patients, particularly when combined with physical rehabilitation [5]. In addition to music, healing frequencies and binaural beats are two other broad categories of auditory stimuli that can serve as noninvasive therapeutic modalities for neurorehabilitation.

Healing frequencies, such as 432 Hz and 528 Hz, may have the potential to support neurorehabilitation. Specific frequencies can promote relaxation, reduce anxiety, and lower cortisol levels, fostering an environment conducive to recovery [6,7]. While direct evidence of their efficacy in TBI and stroke rehabilitation is limited, their capacity to modulate stress and enhance mood presents a promising adjunctive therapy.

Binaural beats, an auditory phenomenon created when tones of slightly different frequencies are played in each ear, have also been shown to facilitate relaxation, improve focus, and enhance cognitive performance. For example, beats at 10 Hz improved cognitive function and attention in university workers and students [8]. Binaural beats may complement traditional rehabilitation approaches by supporting cognitive recovery and emotional well-being.

Together, these findings underscore the significant therapeutic potential of music, healing frequencies, and binaural beats in TBI and stroke rehabilitation. By combining historical insights with modern scientific research, these auditory interventions present an opportunity to improve motor, cognitive, and emotional recovery, offering hope for effective and low-cost patient rehabilitation strategies.

## Review

Methods

This comprehensive review was designed to synthesize current knowledge on the use of music therapy, healing frequencies, and binaural beats for rehabilitation in patients with TBI and stroke. The goal was to integrate findings from diverse study designs, ranging from historical reports and foundational experimental studies to contemporary clinical trials and emerging technologies, in order to outline underlying mechanisms, potential clinical benefits, and future directions for research and practice.

Search Strategy

A literature search was conducted using electronic databases for biomedical research, namely PubMed, MEDLINE, and Google Scholar. The search employed combinations of keywords and MeSH terms related to auditory interventions and neurorehabilitation, including “music therapy,” “healing frequencies,” “binaural beats,” “traumatic brain injury,” “stroke,” and “neuroplasticity.” Particular emphasis was placed on study samples/populations with patients suffering from TBI or stroke. This broad approach allowed for the inclusion of clinical trials, observational studies, case reports, qualitative studies, historical accounts, theoretical models, and reviews.

Selection Criteria

Sources were included if they addressed one or more of the following: (1) the application of music therapy, specific sound frequencies (e.g., 432 Hz, 528 Hz), or binaural beats in the context of TBI or stroke; (2) outcomes related to motor function, cognitive rehabilitation, emotional well-being, or physiological indicators of neural repair and neuroplasticity; and/or (3) mechanistic insights into how auditory stimuli engage neural substrates, such as sensorimotor pathways, limbic circuits, cortical networks, and neurotrophic factor release. No restrictions were placed on study design, and both quantitative and qualitative data were considered. All authors evaluated the validity and relevance of the included studies.

Ethics Statement

No direct patient participation nor primary human data collection was involved in any component of the current study. Therefore, no ethics board approval was required. Otherwise, all published materials cited in the present study were presumed to have obtained ethics approvals when necessary from their respective institutions.

Mechanisms of auditory processing: anatomy and physiology

The Anatomy

The auditory system is a sophisticated network designed to detect, process, and interpret sound. It begins in the peripheral auditory system, where sound waves are collected by the pinna, transmitted through the external auditory canal, and converted into mechanical vibrations at the tympanic membrane. These vibrations are amplified by the ossicles in the middle ear and transmitted to the cochlea in the inner ear. Within the cochlea, mechanosensitive hair cells in the organ of Corti translate sound vibrations into neural signals, organized tonotopically based on frequency [9].

These signals travel through the ascending auditory pathways, starting with the auditory nerve, which transmits information to the cochlear nuclei in the brainstem. The superior olivary complex integrates binaural input for sound localization. Signals then reach the inferior colliculus in the midbrain for reflexive and integrative processing before being relayed to the medial geniculate body of the thalamus, which acts as a relay to the auditory cortex [10].

In the primary auditory cortex, located in Heschl’s gyrus, sounds are processed for fundamental attributes such as frequency and intensity. Secondary and association areas, including the superior temporal gyrus, support complex auditory functions such as speech and music perception [11]. The auditory system also interacts with the limbic system, linking auditory stimuli to emotional responses and memory processing [12].

Auditory evoked potentials (AEPs) are a critical clinical tool for assessing the intactness of the auditory system. AEPs are measured with electrodes placed on the scalp, where auditory stimuli generate waveforms that represent evoked responses in different areas of the brain. This technique provides a comprehensive evaluation of the auditory system, from the ear through to the cortical structures responsible for auditory signal transduction and perception [13].

Advancing this understanding, Li et al. [14] recorded signals from 553 cortical electrodes placed over the auditory cortices of nine participants during a listening task. These recordings were then compared to artificial deep neural networks optimized for speech processing when administered the same auditory stimulus. They found strong correlations between the architecture of the artificial deep neural network and the biological pathways of the ascending auditory system, specifically the auditory nerve, inferior colliculus, Heschl’s gyrus, and superior temporal gyrus. These results suggest that artificial deep neural networks may function similarly to the human auditory system. The integration of deep neural network models into auditory neuroscience research represents a burgeoning field that could provide more sophisticated computational frameworks for using AEPs to evaluate the auditory system.

Beyond these primary pathways, the limbic system, including the amygdala and hippocampus, links auditory stimuli to emotional and memory-related responses, accounting for the emotional impact and autobiographical recall often evoked by music [12]. The prefrontal cortex further modulates attention and decision-making related to auditory stimuli, such as selectively focusing on specific sounds in a noisy environment [15].

The auditory system demonstrates remarkable neuroplasticity, allowing it to adapt to injuries, sensory deprivation, or training. This adaptability is critical in rehabilitation for TBI and stroke, where reorganization of auditory pathways can facilitate recovery and compensate for damaged regions [16]. Disruptions in any segment of the auditory system can lead to deficits in perception and processing, underscoring the clinical significance of understanding these pathways to design targeted therapies, such as music therapy and brainwave entrainment.

By elucidating the neuroanatomy of auditory perception, researchers and clinicians can better understand how auditory interventions influence recovery processes, highlighting their potential in neurorehabilitation for TBI and stroke patients.

The Physiology: Neuroplasticity and Recovery after Brain Injury

One of the primary ways in which auditory experiences facilitate neuroplasticity is through the activation of motor and sensory networks. Rhythmic auditory stimulation (RAS) has been shown to engage the sensorimotor cortex, which helps enhance motor function after brain injury. Research by Thaut et al. [17] demonstrated that rhythmic cues can synchronize with motor output, resulting in improved gait and coordination in stroke patients. The rhythmic component of auditory experiences creates a continuous temporal structure that engages the motor areas, promoting reorganization and adaptation in neural circuits involved in movement.

Studies have also shown that the auditory system has direct connections with the motor system via corticospinal tracts, suggesting that auditory input can influence motor control directly. By stimulating auditory pathways, RAS helps establish new synaptic connections between the auditory and motor cortices, enhancing motor recovery and coordination [18].

Auditory experiences, especially music therapy, have been shown to promote synaptogenesis and dendritic growth in brain regions affected by TBI and stroke. Altenmüller and Schlaug [19] found that musical training leads to increased dendritic branching in the motor and auditory cortices, contributing to improved sensorimotor integration. These structural changes are a direct result of auditory-induced activation, which encourages neuronal growth and connectivity in response to stimulation.

Listening to music also stimulates the release of neurotrophic factors, such as brain-derived neurotrophic factor (BDNF) and nerve growth factor (NGF). These molecules play a critical role in synaptic plasticity, promoting the growth and survival of neurons, particularly in areas of the brain damaged by injury [20]. The release of these factors enhances the brain's capacity to form new synaptic connections, thereby aiding recovery and facilitating functional improvements.

Auditory interventions can also influence neuroplasticity by modulating cortical excitability and entraining brainwaves to specific frequencies. Techniques such as listening to binaural beats can entrain brainwaves, which may influence cortical activity and promote recovery after brain injury [21]. For instance, alpha and theta frequencies are associated with relaxation and focus, potentially facilitating the brain's readiness to engage in rehabilitative tasks. This modulation of cortical excitability supports neuroplastic changes by optimizing the environment for learning and adaptation.

Brainwave entrainment involves the use of RAS to synchronize brainwave frequencies with external auditory beats, thereby enhancing the brain's capacity to reorganize itself [22]. By inducing specific brainwave states, such as alpha (associated with relaxation) or gamma (associated with heightened cognitive function), brainwave entrainment can help optimize conditions for neuroplasticity and promote functional recovery.

Tegeler et al. [23] showed that auditory stimulation can promote hemispheric synchronization and reduce cortical inhibition, enabling the damaged hemisphere to become more responsive to rehabilitation efforts. This effect enhances the potential for functional reorganization, which is crucial for recovery after brain injury.

The biophysical properties of sound play a fundamental role in facilitating neuroplastic changes. Sound waves exert mechanical pressure on cells, which can influence cellular activity and gene expression. The process of mechanotransduction, where mechanical signals are converted into biochemical signals, allows auditory stimuli to impact cellular function. Studies have shown that sound vibrations can stimulate ion channels on the cell membrane, leading to changes in intracellular calcium levels and the activation of signaling pathways involved in neuroplasticity.

At the molecular level, auditory stimulation can influence the expression of genes associated with synaptic plasticity and neuroprotection. For example, music therapy has been shown to upregulate the expression of genes such as BDNF and FOS, which are involved in promoting synaptic growth and neuronal survival [24]. These molecular changes contribute to the reorganization of neural networks, enhancing the brain's capacity to recover after injury.

Music has a unique ability to engage emotional and reward circuitry in the brain, which is important for driving neuroplasticity. The dopaminergic system, which is involved in reward processing, is activated by pleasurable music listening [25]. Dopamine release not only improves mood and motivation, which are essential for participation in rehabilitation, but also plays a key role in synaptic plasticity by enhancing long-term potentiation - a mechanism that underlies learning and memory [26].

Emotional arousal triggered by music can also facilitate the formation of new neural connections. The amygdala, which processes emotions, interacts with the hippocampus during music listening, supporting memory formation and retention. This interaction is particularly beneficial for individuals with TBI or stroke, as it enhances cognitive function and emotional regulation, both of which are crucial for overall recovery [27].

Auditory experiences are inherently bilateral, involving both hemispheres of the brain. This bilateral activation is particularly useful for stroke rehabilitation, where one hemisphere may be damaged. Music therapy encourages the non-injured hemisphere to compensate for lost functions by strengthening neural connections and promoting cortical reorganization [28]. This process, known as interhemispheric transfer, helps balance activity between the hemispheres and supports functional recovery in patients with unilateral brain damage.

Furthermore, Schlaug et al. [29] showed that melodic intonation therapy, which uses musical elements to engage the right hemisphere, can improve language function in stroke patients with left hemisphere damage. This therapy facilitates the recruitment of perilesional areas and homologous regions in the non-injured hemisphere, supporting recovery through adaptive neuroplasticity.

Auditory experiences, including music therapy, RAS, binaural beats, and brainwave entrainment, play a significant role in inducing neuroplastic changes following TBI and stroke. By activating motor and sensory networks, enhancing synaptogenesis, modulating cortical excitability, engaging emotional and reward systems, promoting bilateral cortical reorganization, and leveraging the biophysical properties of sound, these interventions facilitate recovery and improve functional outcomes for patients with brain injuries. The mechanisms underlying these neuroplastic changes provide a promising foundation for developing effective auditory-based rehabilitation strategies to enhance recovery and quality of life in individuals with TBI and stroke.

Therapeutic effects of music on traumatic brain injury and stroke

Several studies have highlighted the multifaceted benefits of music therapy in rehabilitating patients with TBI and stroke, emphasizing its role in improving motor function, cognitive capacity, and emotional well-being. These benefits are supported by neuroimaging and clinical data that reveal changes in brain connectivity and neuroplasticity. A crossover randomized controlled trial (RCT) involving 40 patients with TBI demonstrated significant improvements in executive function among participants who received music therapy during the initial three months of a six-month follow-up period [30]. Follow-up studies using functional magnetic resonance imaging (fMRI) revealed that music therapy-induced cognitive improvements were underpinned by changes in within- and between-network connectivity, particularly between the frontal and parietal regions associated with music processing [31]. A secondary analysis of diffusion MRI results from this RCT further supported these findings, showing structural changes in white matter connectivity that facilitated executive function recovery, structural connectivity changes, neural mechanisms of recovery, and cognitive rehabilitation [32].

In addition to these findings, systematic reviews have provided broader insights into the role of music therapy in cognitive rehabilitation. Alashram et al. [33] systematically reviewed five studies involving 122 patients with TBI and concluded that music therapy can improve executive function. However, evidence for its effects on memory and attention remains limited, highlighting the need for further targeted research in these areas. Regarding motor function, a systematic review and meta-analysis by Ghai [34] evaluated the impact of music therapy on gait in patients with TBI and spinal cord injuries. This review included six studies on TBI and found that music therapy led to moderate improvements in spatiotemporal aspects of gait, such as stride length and cadence. Similarly, Mishra et al. [35] conducted a systematic review and meta-analysis pooling data from six studies, which demonstrated significant improvements in stride length and executive function outcomes in patients undergoing music therapy.

Music therapy has also been shown to be beneficial for stroke rehabilitation. A study examined the role of music therapy in stroke rehabilitation and found that it could enhance limb motor exercise training, promote recovery of speech function, improve dysphagia, and mitigate cognitive impairments [36]. These findings underscore the holistic benefits of music therapy, particularly its ability to address the multifactorial challenges faced by stroke patients.

Altogether, the mechanisms underlying the therapeutic effects of music are diverse, including enhanced neural connectivity within cognitive networks and between brain regions, as well as improvements in executive function, memory, and motor function. Psychologically, music also reduces anxiety and enhances emotional well-being, further feeding into improved recovery. Music therapy is a versatile and effective intervention for rehabilitating cognitive, motor, and emotional functions in TBI and stroke patients. Future research should focus on standardizing therapy protocols, exploring long-term outcomes, and understanding individual variability in responses to music therapy.

Healing frequencies

The concept of healing frequencies, specifically 432 Hz, 528 Hz, 639 Hz, 741 Hz, and 852 Hz, has gained attention in recent years as a potential non-invasive modality to support recovery from TBI and stroke. Healing sound frequencies have been studied for their potential therapeutic effects in promoting neuroplasticity, reducing inflammation, enhancing motor function, improving cognitive recovery and emotional well-being, and facilitating cellular repair, particularly in the context of TBI and stroke recovery. The evidence surrounding these claims, while still emerging, suggests a promising adjunctive role for frequency-based interventions in neurorehabilitation.

Associated with natural harmonics, 432 Hz is suggested to enhance emotional balance and mental clarity. Research indicates exposure to 432 Hz-tuned music reduces anxiety and perceived stress, potentially by modulating neural oscillatory synchronization, supporting an environment conducive to neuroplasticity [37]. Reduced anxiety may indirectly facilitate cognitive rehabilitation by improving patient engagement and sleep quality [38].

Colloquially termed the "miracle frequency," 528 Hz is purported to support cellular health and neurogenesis. Animal studies demonstrate that exposure to 528 Hz reduces oxidative stress in neural tissue, improves mitochondrial function, and increases brain testosterone levels-conditions beneficial for cellular resilience and plasticity [39]. Human studies confirm reduced cortisol and improved mood after exposure to 528 Hz, suggesting stress-reducing effects that could indirectly support neural recovery [40].

While the 639 Hz, 741Hz, and 852 Hz frequencies have also been posited to improve emotional regulation, modulate neuroinflammatory responses, and enhance mental clarity, there is currently little direct empirical evidence supporting these individual frequencies.

While evidence supporting specific frequencies is evolving, general music therapy, especially RAS, has strong empirical backing for motor, cognitive, and emotional rehabilitation post-stroke and TBI [41,42]. Frequency-based interventions, particularly 432 Hz and 528 Hz, may indirectly enhance patient engagement, reduce anxiety, and foster favorable neurophysiological conditions for recovery. As adjunct therapies, these modalities carry low risk, are easily integrated into clinical practice, and offer potential synergistic effects alongside traditional neurorehabilitation strategies.

Further robust research, particularly RCTs, is necessary to validate specific claims about healing frequencies. Currently, frequency-based interventions should be considered supportive therapies rather than primary treatments. Clinicians may employ these frequencies as adjuncts to standard neurorehabilitation, focusing primarily on patient comfort, relaxation, and enhanced therapeutic engagement.

Binaural beats

Binaural beats are an auditory illusion perceived when two tones of slightly different frequencies are presented separately to each ear through stereo headphones or speakers. The brain detects the frequency difference between these tones and generates a third, perceived sound-a "beat" that represents the frequency difference. For instance, if a 440 Hz tone is played in the right ear and a 430 Hz tone in the left, the brain perceives a beat frequency of 10 Hz.

The perception of binaural beats occurs in the superior olivary complex of the brainstem, where the auditory signals from both ears are combined. The brain interprets this difference in frequencies as a rhythmic "beating" effect, which has been suggested to influence brainwave activity.

Binaural beats can potentially entrain brainwave activity by influencing neural oscillations. When individuals listen to binaural beats, their brainwaves tend to synchronize with the frequency of the beats, a phenomenon known as brainwave entrainment. Studies have demonstrated changes in electroencephalography (EEG) patterns corresponding to the frequency of the binaural beats being used. For instance, binaural beats in the alpha range (8-14 Hz) have been associated with increased relaxation, while those in the beta range (14-30 Hz) have been linked to heightened alertness and focus [43].

Binaural beats have been explored for their effects on arousal states, such as stress, anxiety, and alertness. Some studies suggest that listening to beats in different frequency ranges can help modulate emotional and physiological arousal. For instance, theta binaural beats (4-8 Hz) have been shown to promote relaxation, while beta beats (14-30 Hz) can increase arousal and focus. These effects are often evaluated through self-report questionnaires and physiological markers, including heart rate variability [44]. A recent study by Shakya et al. [45] found that exposure to 40 Hz binaural beats enhanced mood and cognitive performance in medical students, while also reducing anxiety during cadaveric dissections. Binaural beats have also been effective in improving sleep quality and reducing anxiety among general volunteers, the elderly, women with obsessive-compulsive disorder, and those experiencing pain [22,46-48].

Research exploring the potential of binaural beats to aid in neural repair following TBI and stroke is still in its early stages. The general hypothesis is that specific frequencies of binaural beats may stimulate neuroplasticity and facilitate functional recovery in patients with brain injuries by altering arousal states and brainwave activity. Enhanced relaxation and focused arousal could help optimize conditions for neuronal regeneration and functional recovery, such as improving sleep quality and reducing stress, both of which are critical for recovery. Studies on music therapy and auditory entrainment have indicated potential benefits for cognitive and motor rehabilitation in stroke patients, and researchers are investigating whether binaural beats could provide similar effects [49].

Socioeconomic impact of auditory interventions

Auditory interventions, such as music therapy, healing frequencies, and binaural beats, are promising approaches for enhancing the rehabilitation of patients with TBI and stroke. However, accessibility and affordability are critical factors that determine the feasibility of these interventions for patients from diverse socioeconomic backgrounds. Studies indicate that while music therapy can be cost-effective compared to other therapeutic modalities, disparities in access to such services persist due to geographic, economic, and insurance-related barriers [50]. Rural and underserved urban areas often lack trained music therapists and infrastructure to support auditory-based rehabilitation, resulting in limited availability for patients who might benefit the most.

The cost-effectiveness of auditory interventions is supported by the relatively low resource requirements. Music therapy, for instance, can be administered in group settings, reducing per-patient costs [51]. Furthermore, digital platforms and mobile applications now make it possible for patients to access guided auditory interventions at home. Despite this, initial costs for technology and the digital divide remain challenges that disproportionately affect lower-income populations, highlighting the need for subsidized or publicly funded initiatives to ensure equitable access.

The integration of auditory interventions into rehabilitation protocols for TBI and stroke patients has the potential to significantly reduce healthcare costs by improving rehabilitation outcomes and shortening hospital stays. A study by Thaut et al. [52] showed that patients who received RAS demonstrated quicker functional recovery, leading to reduced lengths of stay in inpatient rehabilitation facilities. Shorter rehabilitation periods translate to reduced healthcare costs, as patients require fewer intensive care days and less prolonged use of rehabilitation services.

Additionally, the use of music therapy has been associated with improved patient satisfaction and decreased reliance on pharmacological interventions for managing symptoms like anxiety and depression [53]. This not only reduces medication costs but also minimizes the side effects and risks associated with prolonged medication use. The cumulative effect of these benefits points to a more cost-efficient model of care that has the potential to alleviate the financial burden on healthcare systems, especially when serving an aging population with a growing prevalence of stroke and brain injuries.

Cultural perspectives play a significant role in the acceptance and effectiveness of auditory interventions. For instance, in cultures where music is associated with spirituality and healing, there may be a higher level of acceptance and engagement with music therapy. Conversely, some cultures may be skeptical of non-conventional therapies, limiting the reach and effectiveness of these interventions unless they are introduced in culturally sensitive ways [54].

The use of binaural beats and healing frequencies in rehabilitation also reflects cultural and community attitudes toward alternative healing practices. Le Scouarnec et al. [55] found that patients who believed in the therapeutic potential of sound frequencies were more likely to report positive outcomes, highlighting the importance of aligning interventions with patient beliefs. Therefore, it is essential to consider cultural contexts when designing and implementing auditory rehabilitation programs, ensuring that interventions are tailored to meet the needs and expectations of diverse patient populations.

To realize the full potential of auditory interventions in neurorehabilitation, supportive policies are essential. Policymakers must recognize the value of cost-effective therapies like music therapy and incorporate them into standard rehabilitation protocols. Evidence suggests that integrating these therapies can lead to better long-term outcomes, reduce readmission rates, and ultimately lower healthcare expenditures [56]. Therefore, there is a need for policies that incentivize healthcare providers to include music therapy and other auditory interventions as part of a comprehensive rehabilitation strategy.

Insurance coverage is another critical factor in promoting the widespread adoption of auditory interventions. Currently, coverage for music therapy varies significantly, with many patients unable to afford these services out of pocket. Advocacy for broader insurance coverage and public funding could help bridge this gap, making these effective therapies accessible to all patients, regardless of their financial situation. Furthermore, funding for training music therapists and expanding community-based rehabilitation programs would help address the existing disparities in access, particularly in underserved regions.

The socioeconomic impact of auditory interventions in TBI and stroke rehabilitation is profound, with the potential to enhance accessibility, reduce healthcare costs, and address cultural needs within rehabilitation settings. While auditory interventions are cost-effective and beneficial, disparities in access and cultural acceptance present significant challenges that must be addressed through targeted policy initiatives. By supporting the integration of these therapies into mainstream healthcare, policymakers can contribute to a more inclusive and effective rehabilitation system that meets the diverse needs of TBI and stroke patients.

Future directions

The potential of auditory interventions in TBI and stroke rehabilitation lies in advancing technologies, personalizing therapies, and fostering interdisciplinary collaboration. The future of auditory interventions for TBI and stroke rehabilitation is grounded in advancements in neuroimaging, personalized therapies, and interdisciplinary integration. Technologies such as fMRI, diffusion tensor imaging, and magnetoencephalography allow precise mapping of neuroplastic changes and functional connectivity during auditory interventions, while biomarkers like BDNF, NGF, and cytokines offer measurable indicators of neural repair [18]. Artificial intelligence and machine learning can tailor therapies like music and binaural beats to patient-specific profiles, with real-time neurofeedback enhancing therapeutic responsiveness [5]. Emerging technologies, including virtual and augmented reality, combine auditory stimuli with immersive environments to optimize engagement, and wearable devices alongside mobile health applications extend accessibility and adherence [14].

Long-term and large-scale studies are essential to validate these therapies, ensuring they reduce long-term disability and improve quality of life across diverse populations, with a focus on addressing gender and age-specific differences [3]. Integration into multimodal rehabilitation protocols, such as combining auditory therapies with physical or cognitive-behavioral interventions, could yield synergistic benefits, while ethical frameworks must address accessibility for resource-limited settings and ensure informed consent [17]. Finally, mechanistic research into mechanotransduction, mitochondrial function, and inflammation pathways will deepen understanding, while public awareness campaigns and interdisciplinary collaboration will drive the adoption of these innovative, evidence-based practices [14].

## Conclusions

Auditory-based therapies, including music therapy, healing frequencies, and binaural beats, offer a promising and cost-effective avenue for enhancing neurorehabilitation in patients recovering from TBI and stroke. These interventions engage multiple neural pathways, facilitating neuroplasticity, motor recovery, cognitive rehabilitation, and emotional well-being. Music therapy, particularly through RAS, has demonstrated efficacy in improving motor coordination and executive function, while healing frequencies and binaural beats hold potential for modulating arousal states, reducing anxiety, and supporting neural repair. Advances in neuroimaging and electrophysiology continue to elucidate the underlying mechanisms, reinforcing the role of auditory stimulation in promoting structural and functional brain reorganization.

Despite preliminary evidence supporting the benefits of auditory interventions, limitations such as methodological variability, heterogeneity in patient responses, and a lack of large-scale clinical trials underscore the need for further research. Future studies should focus on clinical and long-term outcomes, particularly the sustained impact of auditory interventions on functional independence and quality of life. Standardization of therapy protocols, optimization of individualized treatment approaches, and integration with conventional rehabilitation modalities will be essential for maximizing clinical impact. Emerging technologies, including real-time neurofeedback, artificial intelligence-driven personalization, and immersive virtual and augmented reality applications, may further enhance the precision and accessibility of these therapies. 
